# Coronary Microvascular Dysfunction and Lipid Molecules: Pathophysiological Mechanisms, Clinical Assessment, and Therapeutic Implications

**DOI:** 10.3390/jpm16050254

**Published:** 2026-05-06

**Authors:** Abdelrahman Hafez, Juan M. Farina, Kamal Awad, Milagros Pereyra Pietri, Isabel G. Scalia, Hesham Sheashaa, Fatmaelzahraa E. Abdelfattah, Mahshad Razaghi, Sherif Ahmed, Ramzi Ibrahim, David Simper, Steven J. Lester, Balaji Tamarappoo, Chadi Ayoub, Reza Arsanjani

**Affiliations:** Department of Cardiovascular Medicine, Mayo Clinic, Phoenix, AZ 85054, USA; hafez.abdelrahman@mayo.edu (A.H.); farina.juanmaria@mayo.edu (J.M.F.); awad.kamal@mayo.edu (K.A.); pereyra.milagros@mayo.edu (M.P.P.); scalia.isabel@mayo.edu (I.G.S.); abdelfattah.fatmaelzahraa@mayo.edu (F.E.A.); razaghi.mahshad@mayo.edu (M.R.); ahmed.sherif@mayo.edu (S.A.); ibrahim.ramzi@mayo.edu (R.I.); simper.david@mayo.edu (D.S.); lester.steven@mayo.edu (S.J.L.); tamarappoo.balaji@mayo.edu (B.T.); ayoub.chadi@mayo.edu (C.A.)

**Keywords:** coronary microvascular dysfunction (CMD), ischemia with non-obstructive coronary arteries (INOCA), lipid metabolism, low-density lipoprotein (LDL), lipoprotein(a), triglycerides, endothelial dysfunction, oxidative stress, statins, apolipoprotein B (ApoB), coronary flow reserve (CFR)

## Abstract

Coronary microvascular dysfunction (CMD) has emerged as a crucial contributor to cardiovascular morbidity and mortality, particularly in patients with ischemia and non-obstructive coronary arteries (INOCA). The condition arises from a complex interplay of structural and functional abnormalities within the small coronary vessels, driven by underlying molecular mechanisms including endothelial nitric oxide synthase (eNOS) uncoupling, oxidative stress, and chronic inflammation. Lipid metabolism plays a central role in this pathology, especially in the setting of elevated low-density lipoprotein cholesterol (LDL-C). Furthermore, the protective capacity of high-density lipoprotein (HDL) is increasingly understood to depend on its functionality rather than absolute levels, as it can become dysfunctional and pro-inflammatory in pathological states. Emerging evidence has identified lipoprotein(a) [Lp(a)] and triglyceride-rich lipoproteins as significant, independent contributors to microvascular injury. Comprehensive clinical assessment of microvascular dysfunction therefore requires integration of advanced lipid profiling, including apolipoprotein B (ApoB), [Lp(a)], and the triglyceride-glucose (TyG) index with invasive and non-invasive measures of coronary flow reserve to more precisely stratify risk. In this narrative review, we synthesize current observational, mechanistic, and early interventional data linking diverse lipid phenotypes to coronary microvascular dysfunction. We propose a concept of lipid-driven CMD endotypes, such as ApoB-/particle overload, dysfunctional HDL, Lp(a)-mediated risk, and metabolic/TyG-high states, and map these to a practical, mechanism-informed management framework. While intensive LDL-C lowering with high-intensity statins and combination therapy remains guideline-directed care for high-risk patients, evidence for dedicated microvascular benefit from newer lipid and cardiometabolic agents is still largely hypothesis-generating. A personalized approach that aligns lipid phenotyping, CMD endotyping, and existing guideline-based therapies may help refine risk assessment and inform future trials.

## 1. Introduction

Coronary microvascular dysfunction (CMD) affects the coronary vessels with diameters less than 500 μm and has emerged as a significant contributor to cardiovascular morbidity and mortality [1,2]. Unlike epicardial coronary artery disease, microvascular dysfunction often presents with normal or non-obstructive coronary angiography, leading to the clinical syndrome of ischemia with non-obstructive coronary arteries (INOCA) [3]. Recent studies demonstrate that CMD is associated with a threefold increased risk of major adverse cardiovascular events and represents the primary mechanism responsible for angina and ischemia in patients with non-obstructive coronary artery disease [4,5].

The relationship between lipid metabolism and microvascular function is multifaceted, involving complex interactions between different lipoprotein particles, endothelial function, oxidative stress, and inflammatory pathways [6,7]. Understanding these associations is crucial for developing targeted therapeutic strategies and improving clinical outcomes in patients with microvascular disease. While prior authoritative reviews have expertly detailed the broad pathophysiology of CMD across various cardiac conditions [8] and summarized key clinical takeaways for practitioners [9], a dedicated, concept-driven review that centers specifically on lipid and lipoprotein metabolism from mechanism to management is currently lacking. In this narrative review, we do not attempt a formal, systematic or quantitative synthesis. Instead, we performed focused searches of major biomedical databases, including PubMed, Embase, Cochrane, and Web of Science, emphasizing studies on coronary microvascular dysfunction, lipid biology, diagnostic assessment, and therapeutic strategies. Priority was given to recent, clinically relevant studies, while seminal earlier reports were included when foundational. Based on this approach, we integrate observational and mechanistic evidence to propose a lipid-focused CMD framework, highlighting how distinct lipid phenotypes, including dysfunctional HDL, Lp(a), triglyceride-rich lipoproteins, and TyG-defined metabolic dysfunction, may define testable CMD endotypes and inform hypothesis-generating, phenotype-guided management strategies.

## 2. Core Mechanisms of Microvascular Injury: Structural and Molecular Alterations

Microvascular dysfunction encompasses both structural and functional abnormalities of the coronary microcirculation [2,5]. Structural remodeling involves changes in vessel architecture, including increased media-to-lumen ratio, reduced capillary density, and alterations in perivascular fibrosis. These changes are primarily driven by chronic inflammation, oxidative stress, and neurohormonal activation [3,10].

Functional abnormalities include impaired endothelium-dependent and independent vasodilation, altered vascular reactivity, and increased microvascular resistance [5,11]. The endothelial glycocalyx, a crucial component of vascular homeostasis, undergoes degradation in response to cardiovascular risk factors, leading to disrupted laminar flow and enhanced platelet-endothelial interactions [5,11].

The pathophysiology of microvascular dysfunction involves several interconnected molecular pathways. Endothelial nitric oxide synthase (eNOS) uncoupling represents a central mechanism, where eNOS produces superoxide anion instead of nitric oxide, exacerbating oxidative stress [8,12]. This process is mediated by oxidative depletion of tetrahydrobiopterin (BH4), substrate deficiency, and accumulation of asymmetric dimethylarginine (ADMA) [12].

Inflammatory pathways, including activation of nuclear factor-κB (NF-κB) and production of pro-inflammatory cytokines, contribute to endothelial dysfunction and microvascular impairment [6,7]. The complement system, neutrophil extracellular traps, and NLRP3 inflammasome activation have been identified as additional inflammatory mechanisms contributing to microvascular dysfunction [7].

### 2.1. Low-Density Lipoprotein (LDL) Cholesterol and Endothelial Damage

Low-density lipoprotein cholesterol (LDL-C) exerts profound effects on microvascular function through multiple mechanisms. LDL particles can penetrate the endothelial barrier and accumulate in the vessel wall, where they undergo oxidative modification [6,13]. Oxidized LDL (oxLDL) directly impairs endothelial function by reducing nitric oxide bioavailability, increasing oxidative stress, and promoting inflammatory responses [7,14].

Previous clinical studies have demonstrated a strong association between elevated LDL-C levels and coronary microvascular dysfunction. Mayala et al. conducted a single-center, hospital-based observational study with 40 patients (20 with CMD and 20 with obstructive CAD), comparing clinical characteristics and biomarkers between these groups. They identified LDL-C as an independent predictor for the development of CMD with an odds ratio of 5.24 [15]. Importantly, the association between high LDL-C and impaired microvascular function has been observed across different patient populations, including those with and without obstructive CAD [16]. For instance, Mangiacapra et al. reported in a cohort of 95 patients that elevated total cholesterol—especially LDL cholesterol—correlated strongly with worse coronary microvascular function. In that study, higher LDL-C levels were significantly associated with increased index of microvascular resistance (IMR), indicating poorer microvascular perfusion despite angiographically unobstructed or minimally obstructed arteries [16].

### 2.2. HDL Cholesterol: Protective Mechanisms and Dysfunction

High-density lipoprotein cholesterol (HDL-C) has traditionally been considered protective against cardiovascular disease, but recent research emphasizes HDL functionality over absolute cholesterol levels [17,18]. HDL particles possess multiple cardioprotective functions, including reverse cholesterol transport, antioxidant effects, anti-inflammatory properties, and direct endothelial protection [17,18].

HDL promotes nitric oxide production by endothelial cells through lysophospholipid-mediated pathways and supports endothelial barrier function [19]. The particle also carries paraoxonase-1 (PON1), an enzyme that protects against LDL oxidation and reduces oxidative stress [19]. Additionally, HDL modulates inflammatory responses by inhibiting nuclear factor-κB activation and reducing the expression of adhesion molecules [20].

Patients with CMD were found to have markedly reduced HDL-C levels compared with those without CMD, underscoring the pivotal role of HDL-C in maintaining coronary microvascular integrity [21]. When HDL-C levels are diminished, these protective mechanisms are compromised—cholesterol clearance from peripheral tissues is reduced, oxidative stress is amplified, and nitric oxide bioavailability is impaired—culminating in endothelial dysfunction, a central mechanism in CMD pathogenesis [22]. Low HDL-C further promotes lipid peroxidation and accelerates vascular injury, setting the stage for microcirculatory disturbances [23]. In clinical evaluation, HDL-C serves as a core indicator of lipid metabolism, and its interplay with triglyceride levels, reflected in the triglyceride-to-HDL-C (TG/HDL-C) ratio, offers a more integrated assessment of metabolic and vascular risk [24]. Elevated TG/HDL-C ratios, driven in part by reduced HDL-C, are strongly associated with insulin resistance, atherosclerotic progression, and adverse cardiovascular outcomes, including myocardial infarction, coronary death, and vascular remodeling following ischemic events [21,24]. This relationship highlights the mechanistic and prognostic significance of HDL-C reduction in the context of CMD.

### 2.3. Lipoprotein(a): An Emerging Risk Factor

Lipoprotein(a) [Lp(a)] is a low-density lipoprotein-like particle consisting of apolipoprotein B-100 covalently bound to apolipoprotein(a) [25,26]. Lp(a) levels are primarily genetically determined and serve as an independent, heritable risk factor for atherosclerotic cardiovascular disease [25,26]. The particle is the preferential carrier for oxidized phospholipids and contributes to vascular inflammation, endothelial dysfunction, and thrombogenicity [25,26].

Emerging evidence suggests that elevated Lp(a) levels are associated with increased prevalence of CMD, even in asymptomatic individuals [27]. The mechanisms underlying this association include enhanced oxidative stress, pro-inflammatory effects, and interference with fibrinolysis due to structural homology with plasminogen [28,29]. Lp(a) is more prone to oxidation than LDL-C and can more readily penetrate endothelial barriers to promote foam cell formation [28,29].

### 2.4. Triglycerides and Metabolic Dysfunction

Elevated triglycerides and triglyceride-rich lipoproteins (TRLs) contribute significantly to microvascular dysfunction through multiple pathogenic mechanisms [30]. The triglyceride-glucose (TyG) index has emerged as a reliable marker of insulin resistance and shows strong associations with coronary microvascular dysfunction [31,32]. In patients with chronic coronary syndrome, the TyG index was identified as an independent predictor of CMD with an odds ratio of 1.436 [32].

Hypertriglyceridemia promotes microvascular dysfunction through several pathways. Elevated triglycerides are associated with insulin resistance, which impairs endothelial function and reduces nitric oxide bioavailability [33]. Free fatty acids released from triglyceride-rich lipoproteins can directly impair myocardial microcirculation, as demonstrated by studies showing reduced myocardial blood flow during lipid infusion [34,35,36].

## 3. Novel Lipid Molecules and Coronary Microvascular Dysfunction

### 3.1. Apolipoprotein B and Particle Number

Apolipoprotein B (ApoB) represents the number of atherogenic particles and may provide superior risk assessment compared to cholesterol concentrations alone [37]. Based on a recent retrospective analysis of 145 CAD patients who underwent rest/dipyridamole-stress dynamic SPECT, higher ApoB levels (>1.28 g/L) were independently associated with reduced MFR and greater CMD severity, with an adjusted odds ratio of 11.78 for MFR < 2.5 compared to patients with ApoB < 1.0 g/L, and demonstrated strong predictive performance for CMD (AUC = 0.87) [38]. ApoB measurement is particularly valuable in patients with metabolic syndrome, diabetes, and hypertriglyceridemia, where discordance between LDL-C and particle number is common [39].

Apolipoprotein B (ApoB) offers an additional layer of lipid-related risk stratification. Elevated ApoB levels, even in patients who have achieved target LDL-C levels, signal underlying particle overload. High ApoB correlates with increased microvascular resistance and is predictive of five-year MACE after adjusting for lipid profiles [40].

### 3.2. Oxidized LDL and Advanced Glycation End Products

Oxidized LDL represents a pathophysiologically relevant biomarker directly involved in microvascular injury [41]. The lectin-like oxidized LDL receptor-1 (LOX-1) mediates cellular uptake of oxLDL and contributes to endothelial dysfunction through activation of inflammatory and oxidative stress pathways [42]. Advanced glycation end products (AGEs) accumulate in diabetes and contribute to microvascular complications through receptor for AGE (RAGE)-mediated inflammation [43].

## 4. Special Populations

In diabetes mellitus, hyperglycemia accelerates advanced glycation end-product (AGE) formation and small-vessel fibrosis. TyG-guided lipid therapy intensification has been shown to reduce microvascular complications, including nephropathy and neuropathy [44,45]. In women and those with myocardial infarction and non-obstructive coronary arteries (MINOCA), CMD is often a key underlying factor. A female predominance in low CFR cases highlights the necessity for sex-specific therapeutic strategies [46,47]. Among patients with autoimmune diseases, tumor necrosis factor-alpha (TNF-α) antagonists in rheumatoid arthritis improve capillary density and nailfold microvascular metrics, reinforcing the link between inflammation and lipid dysregulation [48,49].

## 5. Clinical Assessment of Lipid-Mediated Coronary Microvascular Dysfunction

CMD is prevalent among individuals at intermediate to high cardiovascular risk, with a rate of 78.6% observed in patients with an ASCVD risk score above 7.5% [50]. Importantly, CMD is independently associated with an elevated risk of cardiac mortality, even after adjustment for traditional cardiovascular risk factors in the general population [50,51,52]. These observations underscore the importance of careful risk stratification and phenotype-oriented evaluation in patients with suspected microvascular disease.

Clinical assessment of lipid-mediated CMD should integrate functional evaluation of the coronary microcirculation with both standard and advanced lipid profiling. Invasive coronary function testing, including coronary flow reserve (CFR), index of microcirculatory resistance (IMR), and acetylcholine-based vasoreactivity testing, remains central to the identification and phenotyping of CMD. Non-invasive imaging, particularly quantitative PET-derived myocardial flow reserve (MFR), also provides valuable diagnostic and prognostic information [53,54].

Beyond the standard lipid panel, several biomarkers may refine CMD risk assessment. While HDL-C levels have long been considered protective, contemporary evidence emphasizes HDL functionality over quantity. Multiple meta-analyses have shown that HDL cholesterol efflux capacity (HDL-CEC) is inversely associated with major adverse cardiovascular events, independent of HDL-C levels [55,56]. In addition, patients with endothelial dysfunction exhibit reduced HDL particle concentrations and a loss of large HDL subspecies [57], supporting the concept that qualitative HDL abnormalities may contribute to microvascular risk.

Lipoprotein(a) [Lp(a)] also represents an important component of CMD-oriented lipid assessment. Despite affecting approximately 20% of the global population, Lp(a) testing remains underutilized in clinical practice [58]. Elevated Lp(a) levels (≥125 nmol/L) have been associated with a twofold increase in CMD prevalence [28,29], suggesting that Lp(a) measurement may help identify a genetically mediated high-risk phenotype not captured by the standard lipid panel.

Metabolic biomarkers provide additional insight into CMD risk. The triglyceride-glucose (TyG) index, calculated as Ln[fasting triglycerides × fasting glucose/2], reflects insulin resistance and metabolic dysfunction [59]. A TyG index of ≥9.2 independently predicts vulnerable plaques and coronary slow flow, and meta-analyses suggest that each unit increase in TyG index is associated with a 42% higher risk of in-stent restenosis [60,61,62]. The interaction between TyG and visceral adiposity further highlights the complex metabolic contribution to coronary microvascular disease [63]. The TG/HDL-C ratio may also serve as a practical marker of atherogenic dyslipidemia and insulin resistance in this setting.

**Figure 1:**
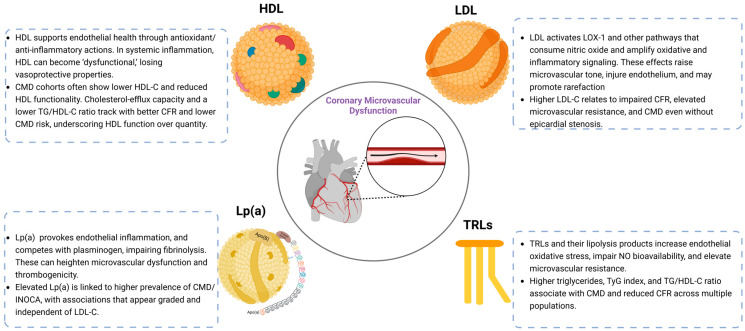
Lipid pathways converging on coronary microvascular dysfunction (CMD).

Taken together, the assessment of lipid-mediated CMD should move beyond conventional cholesterol measurements alone and incorporate advanced lipid biomarkers, metabolic indices, and physiologic measures of coronary microvascular function. Figure 1 and Table 1 summarize the principal lipid-related mechanisms involved in CMD pathophysiology and the corresponding approaches to clinical assessment and monitoring.

## 6. Therapeutic Strategies

Although several lipid-lowering and cardiometabolic therapies may influence mechanisms relevant to coronary microvascular dysfunction, direct evidence for CMD-specific clinical benefit remains limited, and much of the available literature is based on observational data, mechanistic studies, or surrogate physiological endpoints.

### 6.1. Lipid-Lowering Therapies

High-intensity statin therapy represents the cornerstone of lipid management in patients at high atherosclerotic risk who may also harbor CMD [64]. Beyond LDL-C reduction, statins exert pleiotropic effects, including improved endothelial function, anti-inflammatory actions, and enhanced nitric oxide bioavailability, that are plausibly beneficial for the coronary microcirculation [65]. Small mechanistic studies suggest that statins may improve CFR and reduce microvascular resistance in selected populations [66], but these data remain limited, and CMD-specific outcome benefits have not been definitively established.

PCSK9 inhibitors provide potent LDL-C lowering and modest reductions in Lp(a), with robust evidence for macrovascular risk reduction in very-high-risk patients [67]. Preliminary data from trials such as EVOCATION and FITTER suggest possible favorable effects on CFR in selected subgroups, although evolocumab pretreatment did not consistently prevent periprocedural microvascular dysfunction, and residual CMD remained common [68]. Thus, PCSK9 inhibitors should currently be viewed as guideline-directed agents for LDL-C and Lp(a) risk reduction, with any putative microvascular benefits considered exploratory. Ezetimibe offers incremental LDL-C lowering and is well tolerated in combination with statins, and its role in CMD is similarly inferred from its impact on overall cardiovascular risk rather than from dedicated CMD trials [69,70].

### 6.2. Targeted Microvascular Therapies

Calcium channel blockers, particularly those with long half-lives, provide effective symptom relief and improved exercise tolerance in patients with microvascular dysfunction [60]. Ranolazine offers anti-ischemic effects through improved diastolic function and has demonstrated symptom improvement and quality of life benefits [60]. Nebivolol, a beta-blocker with nitric oxide-mediated vasodilatory properties, may provide specific benefits for microvascular function without compromising heart rate response [60]. Renin-angiotensin system inhibitors prevent free fatty acid-induced microvascular dysfunction and provide long-term endothelial protection [60].

### 6.3. Metabolic and Lifestyle Interventions

For patients with type 2 diabetes or cardiometabolic disease, SGLT2 inhibitors and GLP-1 receptor agonists confer substantial cardiovascular and renal benefits that extend beyond glucose control [61,62]. Experimental data and small clinical studies suggest that these agents may favorably influence endothelial function, inflammation, and myocardial energetics, raising the possibility of beneficial effects on the coronary microcirculation [61,62,71]. However, microvascular endpoints have rarely been prespecified, and available CMD data are hypothesis-generating rather than definitive. Accordingly, SGLT2 inhibitors and GLP-1 receptor agonists should be prescribed based on established indications (heart failure, ASCVD, diabetes with high risk), with any potential CMD benefit considered an attractive but as yet unproven advantage. Metformin remains foundational therapy for type 2 diabetes and has been associated with improved endothelial function, but its impact on CMD-specific outcomes also requires further study [71].

Exercise training represents a powerful intervention for improving microvascular function through enhanced nitric oxide bioavailability, angiogenesis, and reduced oxidative stress [72]. Structured exercise programs demonstrate improvements in coronary flow reserve and quality of life measures [73]. Therapeutic strategies and management approaches for coronary microvascular dysfunction are summarized in Table 2.

## 7. Current Guidelines and Clinical Recommendations

### 7.1. 2024. ESC Guidelines Updates

CMD is prevalent among people with moderate to high cardiovascular risk and is independently associated with an increased likelihood of cardiac mortality, even after accounting for traditional cardiovascular risk factors [50,51,52]. The 2024 European Society of Cardiology guidelines introduce new blood pressure categories and emphasize comprehensive cardiovascular risk assessment, especially for high-risk patients [74]. The guidelines maintain aggressive LDL-C targets below 1.4 mmol/L for very high-risk patients while recognizing the importance of non-HDL cholesterol and apolipoprotein B as secondary targets [75,76].

Recent updates emphasize the role of combination therapy for achieving lipid targets, with increased utilization of ezetimibe and PCSK9 inhibitors in appropriate patients [75,77]. The guidelines acknowledge the emerging role of lipoprotein(a) as an independent risk factor and recommend screening in intermediate-risk patients [75,77].

### 7.2. Personalized Medicine Approaches

Current evidence supports consideration of phenotype-informed management approaches based on individual patient characteristics and microvascular dysfunction patterns, although prospective validation remains limited [78]. The identification of different CMD endotypes using invasive functional testing allows for targeted therapy selection [79]. High-hyperemic-resistance and low-hyperemic-resistance subtypes require different therapeutic approaches based on underlying pathophysiological mechanisms [2].

#### 7.2.1. Genetic Insights and Pharmacogenomics

Recent genetic studies have begun to uncover single-nucleotide polymorphisms (SNPs) associated with an increased risk of CMD. Variants in genes such as eNOS and CDKN2B-AS1, as well as those regulating endothelin-1, have been implicated in the pathogenesis of microvascular dysfunction [80,81]. Notably, some of these genetic loci are also involved in pathways governing lipid metabolism, suggesting a potential genetic link between dyslipidemia and a predisposition to CMD [80]. The identification of these and other genetic markers may help inform future risk stratification and targeted preventive strategies in susceptible individuals.

Furthermore, the field of pharmacogenomics offers the potential to tailor lipid-lowering therapies to maximize efficacy and minimize adverse effects. It is well-established that there is significant inter-individual variability in the response to statins, the cornerstone of lipid-lowering therapy [82]. Genetic polymorphisms can influence the pharmacokinetics and pharmacodynamics of statins, affecting both the degree of LDL-C reduction and the risk of statin-associated muscle symptoms [82]. For patients with CMD and concomitant dyslipidemia, genotype-guided statin therapy may eventually help optimize treatment outcomes, although this approach is not yet established. For instance, individuals with certain genetic variants may respond differently to lipid-lowering agents or require alternative dosing strategies, though further validation is needed before routine clinical application [83].

#### 7.2.2. Deep Phenotyping with Advanced Lipid and Inflammatory Biomarkers

Moving beyond standard lipid panels towards “deep phenotyping” is crucial for a personalized assessment of CMD. Advanced lipid profiling, or lipidomics, can identify specific lipid signatures associated with microvascular dysfunction that are not captured by routine cholesterol measurements [84,85]. For example, the previous literature has shown that distinct profiles of ceramides and other sphingolipids, which play a role in inflammation and endothelial dysfunction, are associated with an increased risk of cardiovascular events [84,85]. Assessing these novel lipid biomarkers can provide a more granular understanding of an individual’s pathological state and guide more targeted therapeutic interventions [84,85].

In addition to advanced lipid profiling, a comprehensive assessment of inflammatory biomarkers is critical. Given the central role of inflammation in both dyslipidemia and CMD, measuring markers such as high-sensitivity C-reactive protein (hs-CRP), interleukin-6 (IL-6), and lipoprotein-associated phospholipase A2 (Lp-PLA2) can help to stratify risk and monitor response to therapy [86,87].

### 7.3. Clinical Implications and Practice Points

#### 7.3.1. Personalized Therapeutic Mapping of Coronary Microvascular Dysfunction

Clinicians should maintain high clinical suspicion for microvascular dysfunction in patients presenting with angina and non-obstructive coronary arteries, particularly women and patients with diabetes [88]. The triglyceride-glucose index and LDL/HDL ratio provide accessible tools for metabolic risk assessment and early identification of microvascular dysfunction risk [89,90]. Comprehensive lipid assessment should include non-HDL cholesterol, apolipoprotein B, and lipoprotein(a) measurement in appropriate patients [91].

Building on these insights, emerging evidence emphasizes that not all coronary microvascular dysfunction is the same; distinct endotypes, such as endothelial dysfunction with vasospasm, impaired microvascular dilation, structural high-resistance remodeling, metabolic CMD, and Lp(a)-driven disease, carry different pathophysiological mechanisms and therapeutic implications. Recognizing these subtypes may help clinicians move beyond a ‘one-size-fits-all’ approach and support a mechanism-informed, individualized management framework. The following table (Table 3) summarizes how diagnostic phenotyping, biomarker profiles, and lipid metrics may be aligned with potential therapeutic considerations, offering a hypothesis-generating framework for precision-oriented management.

#### 7.3.2. Monitoring and Follow-Up

Regular assessment of symptoms, functional capacity, and lipid parameters is essential for optimizing long-term outcomes [9]. Non-invasive imaging modalities can be used for monitoring treatment response and disease progression [9]. Patient education regarding the chronic nature of microvascular dysfunction and the importance of long-term adherence to medical therapy is crucial for successful management [96].

## 8. Knowledge Gap and Critical Appraisal

The available evidence linking lipid abnormalities to coronary microvascular dysfunction remains heterogeneous and largely observational. Most studies in this review rely on cross-sectional imaging, single-center cohorts, or small mechanistic interventions, with limited adjustment for confounders and substantial variation in how CMD is defined and measured [24,31,32,61,62,71]. As a result, associations between ApoB, triglyceride-rich lipoproteins, TG/HDL-C, TyG, Lp(a), and impaired CFR or elevated IMR should be interpreted as signals of potential mechanistic relevance rather than proof that modifying any single lipid parameter will reverse CMD. Likewise, data on therapeutic interventions (statins, PCSK9 inhibitors, GLP-1 receptor agonists, SGLT2 inhibitors, and lifestyle/exercise programs) are strongest for macrovascular outcomes, whereas microvascular benefits are inferred from surrogate endpoints or small substudies [55,56,57,75,76,77,78,79,80,81,82,83].

From a clinical standpoint, these limitations argue against overextending the current evidence and instead support a cautious, phenotype-informed approach. Advanced lipid profiling (ApoB, non-HDL-C, TG/HDL-C, TyG, and Lp(a)) can help identify patients with high atherogenic and metabolic burden who are already candidates for intensive guideline-directed therapy under contemporary guidelines recommendations [70,87]. In such patients, the recognition of CMD, particularly in women with INOCA/MINOCA, individuals with diabetes or insulin resistance, and those with autoimmune disease, may serve as an additional “red flag” that justifies stricter LDL-C and ApoB targets, combination lipid-lowering strategies, and aggressive cardiometabolic risk modification, without claiming CMD-specific benefit beyond established outcome data.

Practically, clinicians can integrate these insights into three critical steps: (1) maintain a high index of suspicion for CMD in symptomatic patients with non-obstructive CAD or discordant ischemia–anatomy findings, especially in high-risk phenotypes highlighted in this review; (2) use advanced lipid and cardiometabolic indices to refine global risk assessment and to guide intensity of guideline-directed therapy, rather than to drive off-label or unproven CMD-specific interventions; and (3) monitor symptoms, functional capacity, lipid profile, and non-invasive CMD surrogates over time to ensure that treatment decisions remain anchored in both patient-reported outcomes and evolving evidence [96]. In this way, a lipid-centric CMD framework can support thoughtful, individualized care while explicitly acknowledging that definitive microvascular outcome data are still needed and that many of the proposed endotypes and therapeutic links remain hypothesis-generating.

## 9. Conclusions

Coronary microvascular dysfunction represents a pivotal, yet heterogeneous, cardiovascular pathology in which lipid disturbances play a central but not uniform role. Rather than a single entity, CMD appears to comprise multiple lipid-linked endotypes, including ApoB-/particle overload, dysfunctional HDL, Lp(a)-mediated risk, and metabolic/TyG-driven phenotypes, that intersect with sex, diabetes, and inflammatory states. In this narrative review, we synthesize observational, mechanistic, and early interventional evidence to propose a lipid-centric CMD framework that connects these endotypes to practical, phenotype-informed management strategies. Optimal care currently relies on guideline-directed risk factor modification, including intensive LDL-C lowering, blood pressure control, cardiometabolic therapies, and targeted microvascular-directed agents, while recognizing that definitive CMD-specific outcome data for many lipid and cardiometabolic drugs are still lacking. Moving forward, integrating advanced lipid and inflammatory profiling with invasive and PET-based CMD phenotyping, and designing prospective trials that prespecify microvascular endpoints, will be essential to test whether targeting specific lipid phenotypes can truly modify CMD and its clinical consequences. Until such data are available, lipid-focused personalization of CMD management should be viewed as a rigorous, hypothesis-generating paradigm, not as established causal proof.

## Figures and Tables

**Table 1:** Clinical Assessment and Monitoring of Coronary Microvascular Dysfunction.

Assessment Category	Method/Biomarker	Purpose and What It Measures	Clinical Utility in CMD
**Invasive Functional Testing [54]**	Coronary Function Testing (e.g., with Acetylcholine)	Directly assesses endothelium-dependent and independent vasodilation and measures coronary flow reserve (CFR) and index of microcirculatory resistance (IMR).	Gold standard for diagnosing and phenotyping CMD (e.g., high- vs. low-resistance subtypes).
**Non-Invasive Imaging [53]**	Quantitative PET Perfusion	Ratio that normalizes methodological variability; integrates resting and stress flow to reflect coronary vasodilator reserve.	Universal diagnostic cutoff Myocardial Flow Reserve (MFR) < 2.0 for CMD; <1.7 or <1.5 identifies markedly elevated risk of MACE.
**Standard Lipid Panel**	LDL-C, HDL-C, Triglycerides	Measures cholesterol and triglyceride concentrations within lipoproteins.	Foundational for cardiovascular risk assessment; high LDL-C and TG are associated with CMD.
**Advanced Lipid Biomarkers [38]**	Apolipoprotein B (ApoB)	Measures the total number of atherogenic lipoprotein particles (LDL, VLDL, Lp(a)).	Superior risk predictor than LDL-C, especially in metabolic syndrome; correlates with microvascular resistance.
	Lipoprotein(a) [Lp(a)]	Measures the concentration of a distinct, highly atherogenic particle.	Identifies a significant genetic risk factor for CMD, independent of other lipids. Recommended for screening.
**Metabolic Biomarkers**	Triglyceride-Glucose (TyG) Index	A surrogate marker for insulin resistance.	A simple, accessible tool that independently predicts CMD and vulnerable plaques.
	TG/HDL-C Ratio	A marker of atherogenic dyslipidemia and insulin resistance.	Strong association with reduced CFR and is an independent predictor of microvascular dysfunction.

Abbreviations: CMD = Coronary Microvascular Dysfunction; CFR = Coronary Flow Reserve; IMR = Index of Microcirculatory Resistance; PET = Positron Emission Tomography; MFR = Myocardial Flow Reserve; LDL-C = Low-Density Lipoprotein Cholesterol; HDL-C = High-Density Lipoprotein Cholesterol; TG = Triglycerides; ApoB = Apolipoprotein B; VLDL = Very-Low-Density Lipoprotein; Lp(a) = Lipoprotein(a); TyG = Triglyceride-Glucose Index; TG/HDL-C = Triglyceride-to-HDL Cholesterol Ratio; MACE = Major Adverse Cardiovascular Events.

**Table 2:** Therapeutic Strategies and Management of Coronary Microvascular Dysfunction.

Therapeutic Class	Specific Agent(s)	Primary Mechanism of Action in CMD	Key Clinical Benefit
**Lipid-Lowering Therapies**	Statins	↓ LDL-C; Pleiotropic effects: improves endothelial function, ↓ inflammation, ↑ nitric oxide bioavailability.	First-line therapy for risk reduction, with possible benefit on CFR.
	Ezetimibe	Inhibits cholesterol absorption, providing incremental LDL-C reduction.	Used in combination with statins to achieve LDL-C goals.
	PCSK9 Inhibitors	Potently ↓ LDL-C and Lp(a) by increasing LDL receptor recycling.	Significant LDL-C reduction, with possible benefit on CFR.
	Lp(a)-Targeted Therapies	Antisense Oligonucleotides (e.g., Zerlasiran)	Specifically inhibits the production of apolipoprotein(a).
**Targeted Microvascular Therapies**	Calcium Channel Blockers	Causes coronary vasodilation and reduces microvascular spasm.	Symptom relief (angina) and improved exercise tolerance.
	Ranolazine	Anti-ischemic effects through inhibition of the late sodium current, improving diastolic function.	Symptom improvement and enhanced quality of life.
	Nebivolol	Beta-blockade with added nitric oxide-mediated vasodilation.	Reduces heart rate while potentially improving endothelial function.
	RAS Inhibitors (ACEi/ARBs)	Prevent angiotensin II-mediated vasoconstriction and inflammation; protect against free fatty acid-induced dysfunction.	Long-term endothelial protection and blood pressure control.
**Metabolic and Lifestyle Interventions**	SGLT2 Inhibitors/GLP-1 RAs	Glucose-lowering with added anti-inflammatory, anti-fibrotic, and direct cardiovascular benefits.	Reduce cardiovascular events and may improve microvascular function in diabetes.
	Metformin	Enhances insulin sensitivity and improves endothelial function.	Foundational therapy for type 2 diabetes with microvascular benefits.
	Structured Exercise Training	↑ Nitric oxide bioavailability, promotes angiogenesis, ↓ oxidative stress.	Powerful non-pharmacological tool to improve CFR and quality of life.

Abbreviations: ↑, increased/higher than baseline; ↓, decreased/lower than baseline; CMD = Coronary Microvascular Dysfunction; LDL-C = Low-Density Lipoprotein Cholesterol; Lp(a) = Lipoprotein(a); CFR = Coronary Flow Reserve; PCSK9 = Proprotein Convertase Subtilisin/Kexin Type 9; ACEi = Angiotensin-Converting Enzyme Inhibitor; ARB = Angiotensin II Receptor Blocker; RAS = Renin–Angiotensin System; SGLT2 = Sodium–Glucose Cotransporter 2; GLP-1 RA = Glucagon-Like Peptide-1 Receptor Agonist.

**Table 3:** Precision Medicine Approach: Matching CMD Phenotypes with Biomarker and Suggested Treatment Strategies.

CMD Endotype (Phenotyping Anchor)	Typical Testing Modality [53,54,78,92]	Biomarker [53,54,78,92]	Potential First-Line Management Consideration	Possible Add-On Strategies
**Endothelial dysfunction/microvascular spasm [9,54,60]**	ACh: spasm or impaired dilation; variable CFR; symptoms at low ACh dose	Often Lp(a) ↑, ApoB ↑; inflammation signal	Long-acting CCBs; RAS inhibition for endothelial support	Ranolazine for symptoms; consider statin + ezetimibe/PCSK9 to hit LDL-C/ApoB targets; address Lp(a) where high (PCSK9 now; ASO/siRNA emerging)
**Impaired microvascular dilation [64,65,66]**	CFR ↓, MFR < 2.0; IMR elevated or normal	ApoB ↑, sdLDL burden, TG/HDL-C ↑	High-intensity statin ± ezetimibe; ACEi/ARB; exercise prescription	PCSK9 inhibitor if targets unmet; SGLT2i/GLP-1RA in diabetes/metabolic syndrome; Ranolazine for persistent angina
**High-resistance/structural remodeling [16,53,54]**	IMR ↑, minimal vasodilator reserve; PET MFR often <1.7	ApoB ↑; may see TyG ↑	Aggressive LDL/ApoB lowering (statin ± ezetimibe ± PCSK9) and BP control; exercise	Consider cardiac rehab; evaluate for lipidomics/inflammation panel if refractory
**Metabolic CMD (insulin resistance dominant) [31,32]**	Flow depression under stress; slow flow phenomenon	TyG ≥ 9.2, TG/HDL-C ↑, HDL-CEC often low	SGLT2 inhibitor or GLP-1RA (if T2D), statin per risk, weight loss interventions	Intensify statin; add fibrate only for severe hypertriglyceridemia; focus on lifestyle and CRF gains
**Lp(a)-driven risk [26,27,93,94,95]**	May have discordant risk vs. LDL-C; structural/functional changes	Lp(a) ≥ 125 nmol/L	PCSK9 inhibitor (modest Lp(a) lowering) + strict LDL-C/ApoB targets	Consider ongoing trials for ASO/siRNA (e.g., zerlasiran) when available; apheresis in extreme cases

Abbreviations: ↑, increased; ↓, decreased. CMD = Coronary Microvascular Dysfunction; ACh = Acetylcholine; CFR = Coronary Flow Reserve; MFR = Myocardial Flow Reserve; IMR = Index of Microcirculatory Resistance; Lp(a) = Lipoprotein(a); ApoB = Apolipoprotein B; sdLDL = Small Dense Low-Density Lipoprotein; TG/HDL-C = Triglyceride-to-HDL Cholesterol Ratio; TyG = Triglyceride-Glucose Index; HDL-CEC = HDL Cholesterol Efflux Capacity; CCB = Calcium Channel Blocker; RAS = Renin–Angiotensin System; ACEi = Angiotensin-Converting Enzyme Inhibitor; ARB = Angiotensin II Receptor Blocker; PCSK9 = Proprotein Convertase Subtilisin/Kexin Type 9; SGLT2i = Sodium–Glucose Cotransporter 2 Inhibitor; ASO = Antisense Oligonucleotide; siRNA = Small Interfering RNA; CRF = Cardiorespiratory Fitness; T2D = Type 2 Diabetes.

## Data Availability

No new data were created or analyzed in this study. Data sharing is not applicable to this article.

## Abbreviations

The following abbreviations are used in this manuscript:

CMD	Coronary Microvascular Dysfunction
INOCA	Ischemia with Non-Obstructive Coronary Arteries
eNOS	Endothelial Nitric Oxide Synthase
LDL-C	Low-Density Lipoprotein Cholesterol
HDL-C	High-Density Lipoprotein Cholesterol
Lp(a)	Lipoprotein(a)
ApoB	Apolipoprotein B
TyG	Triglyceride-Glucose Index
IMR	Index of Microvascular Resistance
CFR	Coronary Flow Reserve
PET	Positron Emission Tomography
MFR	Myocardial Flow Reserve
MACE	Major Adverse Cardiovascular Events
HDL-CEC	HDL Cholesterol Efflux Capacity
oxLDL	Oxidized Low-Density Lipoprotein
BH4	Tetrahydrobiopterin
ADMA	Asymmetric Dimethylarginine
NF-κB	Nuclear Factor Kappa-B
NLRP3	NOD-Like Receptor Pyrin Domain-Containing 3 (Inflammasome)
PON1	Paraoxonase-1
TRL	Triglyceride-Rich Lipoproteins
sdLDL	Small Dense Low-Density Lipoprotein
EVOCATION	Evolocumab for the Prevention of Coronary Microvascular Dysfunction Trial
FITTER	Evolocumab Clinical Trial (Future Investigation Targeting Triglycerides and Endothelial Reserve)
LOX-1	Lectin-Like Oxidized LDL Receptor-1
AGE	Advanced Glycation End Product
RAGE	Receptor for Advanced Glycation End Products
MINOCA	Myocardial Infarction with Non-Obstructive Coronary Arteries
TNF-α	Tumor Necrosis Factor-Alpha
PCSK9	Proprotein Convertase Subtilisin/Kexin Type 9
ACEi	Angiotensin-Converting Enzyme Inhibitor
ARB	Angiotensin Receptor Blocker
SGLT2	Sodium-Glucose Cotransporter-2
GLP-1 RA	Glucagon-Like Peptide-1 Receptor Agonist
ESC	European Society of Cardiology
ASCVD	Atherosclerotic Cardiovascular Disease
